# A scoping review of comparisons between abstracts and full reports in primary biomedical research

**DOI:** 10.1186/s12874-017-0459-5

**Published:** 2017-12-29

**Authors:** Guowei Li, Luciana P. F. Abbade, Ikunna Nwosu, Yanling Jin, Alvin Leenus, Muhammad Maaz, Mei Wang, Meha Bhatt, Laura Zielinski, Nitika Sanger, Bianca Bantoto, Candice Luo, Ieta Shams, Hamnah Shahid, Yaping Chang, Guangwen Sun, Lawrence Mbuagbaw, Zainab Samaan, Mitchell A. H. Levine, Jonathan D. Adachi, Lehana Thabane

**Affiliations:** 1 Department of Health Research Methods, Evidence, and Impact, McMaster University, Hamilton, ON Canada; 2 St. Joseph’s Healthcare Hamilton, McMaster University, 501-25 Charlton Avenue East, Hamilton, ON L8N 1Y2 Canada; 3 Centre for Evaluation of Medicines, Programs for Assessment of Technology in Health (PATH) Research Institute, McMaster University, Hamilton, ON Canada; 4 Department of Dermatology and Radiotherapy, Botucatu Medical School, Universidade Estadual Paulista, UNESP, São Paulo, Brazil; 5 Faculty of Health Sciences, McMaster University, Hamilton, ON Canada; 6 McMaster Integrative Neuroscience Discovery and Study, McMaster University, Hamilton, ON Canada; 7 Medical Sciences, McMaster University, Hamilton, ON Canada; 8 Integrated Sciences, McMaster University, Hamilton, ON Canada; 9 Psychology, Neuroscience and Behaviour, McMaster University, Hamilton, ON Canada; 10 Arts and Science, McMaster University, Hamilton, ON Canada; 11 Department of Medicine, McMaster University, Hamilton, Canada; 12 Father Sean O’Sullivan Research Centre, St. Joseph’s Healthcare Hamilton, 3rd Floor Martha, Room H325, 50 Charlton Avenue E, Hamilton, ON L8N 4A6 Canada

**Keywords:** Abstract, Scoping review, Inconsistent reporting, Deficiency, Accuracy, Discrepancy, Spin

## Abstract

**Background:**

Evidence shows that research abstracts are commonly inconsistent with their corresponding full reports, and may mislead readers. In this scoping review, which is part of our series on the state of reporting of primary biomedical research, we summarized the evidence from systematic reviews and surveys, to investigate the current state of inconsistent abstract reporting, and to evaluate factors associated with improved reporting by comparing abstracts and their full reports.

**Methods:**

We searched EMBASE, Web of Science, MEDLINE, and CINAHL from January 1st 1996 to September 30th 2016 to retrieve eligible systematic reviews and surveys. Our primary outcome was the level of inconsistency between abstracts and corresponding full reports, which was expressed as a percentage (with a lower percentage indicating better reporting) or categorized rating (such as major/minor difference, high/medium/low inconsistency), as reported by the authors. We used medians and interquartile ranges to describe the level of inconsistency across studies. No quantitative syntheses were conducted. Data from the included systematic reviews or surveys was summarized qualitatively.

**Results:**

Seventeen studies that addressed this topic were included. The level of inconsistency was reported to have a median of 39% (interquartile range: 14% - 54%), and to range from 4% to 78%. In some studies that separated major from minor inconsistency, the level of major inconsistency ranged from 5% to 45% (median: 19%, interquartile range: 7% - 31%), which included discrepancies in specifying the study design or sample size, designating a primary outcome measure, presenting main results, and drawing a conclusion. A longer time interval between conference abstracts and the publication of full reports was found to be the only factor which was marginally or significantly associated with increased likelihood of reporting inconsistencies.

**Conclusions:**

This scoping review revealed that abstracts are frequently inconsistent with full reports, and efforts are needed to improve the consistency of abstract reporting in the primary biomedical community.

## Background

Abstracts of primary research can provide concise information on a study’s purpose, methods, main results and conclusions. It is not uncommon that abstracts are used to aid decision-making, especially when full reports cannot be accessed [1, 2]. However, there is a risk that the reporting of abstracts is inconsistent with their corresponding full reports, which would then distract or mislead readers [3, 4]. Although the EQUATOR (Enhancing Quality and Transparency in Health Research) network has provided some guidelines to enhance the reporting of abstracts [5], adherence to them remains unsatisfactory [6–9]. Some studies assessing the inconsistency between abstracts and full reports have reported striking findings of abstract inaccuracy [10–12]. Nevertheless, there is a lack of summary showing the general mapping of the abstract reporting problem or providing overarching recommendations in primary studies for future research in the literature. Therefore, as part our series on the state of reporting of primary biomedical research [13], we used a scoping review to summarize the evidence from systematic reviews and surveys, in order to investigate the current state of inconsistent abstract reporting, and also to evaluate factors that are associated with improved reporting. This was done by comparing abstracts and their corresponding full reports.

## Methods

We performed and reported our study based on the methodological guidance for the conduct of a scoping review from the Joanna Briggs Institute [14] and the PRISMA (Preferred Reporting Items for Systematic Reviews and Meta-Analyses) guideline [15]. Details on the methods can be found in our protocol [13]. Surveys and systematic reviews were considered eligible if they compared abstracts within full reports with the reports themselves, or if they compared conference abstracts with subsequent full reports emanating from the same study. We did not distinguish between these comparisons in this scoping review.

### Search strategy and study selection

Briefly, we searched EMBASE (Exerpta Medica Database), Web of Science, MEDLINE, and CINAHL (Cumulative Index to Nursing and Allied Health Literature) from January 1st 1996 to September 30th 2016 to retrieve relevant studies, using key descriptors for systematic reviews or surveys, abstracts, and reporting or inconsistency. All reference lists from the included surveys and reviews were also manually searched to assess their eligibility. All the searches were limited to the English language. Studies were excluded if: 1) they were not systematic reviews or surveys; 2) they did not have a study objective of comparing abstracts with full reports; 3) no data were reported on inconsistency between abstracts and full reports; 4) they were in duplicate (and then only one copy was retained); 5) they only published abstracts, letters, editorials or commentaries without full-text articles for further detailed information; or 6) they did not focus on primary studies.

All the screening of titles, abstracts and full-text articles was conducted by two reviewers (IN and YJ) in duplicate and independently. We used the Kappa statistic to quantify the level of agreement between the two reviewers [16]. Discrepancies between the two reviewers were resolved by consensus, or, failing that, a third reviewer (GL) made a final decision.

### Outcome measures

The primary outcome was the level of inconsistency between abstracts and full reports, which was expressed as a percentage (lower percentage indicating better reporting) or categorized rating (such as major/minor difference, high/medium/low inconsistency), as reported by the authors. We also extracted details on inconsistency for the study-validity-related factors including research question or objective, population or sample size, intervention or exposure, comparator, outcome, study duration, study design, statistical analysis, result presentation, result interpretation, and conclusion or recommendation [13]. Secondary outcomes were the factors associated with inconsistent abstract reporting.

### Data collection

Data collection was conducted independently by two reviewers (LA and IN) using a pilot-tested data extraction form. Data extracted were: 1) general characteristics of included systematic reviews or surveys (authors, journal, publication year, study design, field of study, data sources for abstracts and full reports, study search frame, numbers of included abstracts and full reports, study country of primary studies, study sample size in primary studies, and funding information for the systematic reviews or surveys); 2) definitions of inconsistency between abstracts and full reports and the main findings in the included studies; 3) information on inconsistency for the study-validity-related factors; 4) factors related to improved reporting between abstracts and full reports; and 5) authors’ conclusions or recommendations in the included studies. We also collected the terminologies that were used to describe the abstract reporting problem, and their frequencies.

### Quality assessment of included systematic reviews

We assessed the study quality of included systematic reviews based on the AMSTAR (a measurement tool to assess systematic reviews) criteria [17]. Some items, such as item 9 (“Were the methods used to combine the findings of studies appropriate?”) and item 10 (“Was the likelihood of publication bias assessed?”) were not applicable to the included systematic reviews, and these items were therefore excluded from our overall quality score evaluation. We calculated the scores by summing the number of items on AMSTAR that the included systematic reviews met. No study quality assessment was performed for the surveys because there were no validated evaluation tools available.

### Evidence synthesis

We used word clouds to show the frequencies of the terminologies employed to describe the problems identified in abstract reporting. The online program Wordle (www.wordle.net) was used to draw the word clouds, based on input of the terminologies and the numbers of included studies that used them to describe inconsistent abstract reporting. The relative size of the terminologies in the word clouds corresponded to the frequency of their use. We used medians and interquartile ranges to describe the level of inconsistency across studies. Evidence from the included systematic reviews or surveys was summarized qualitatively, but not quantitatively.

## Results

**Fig. 1:**
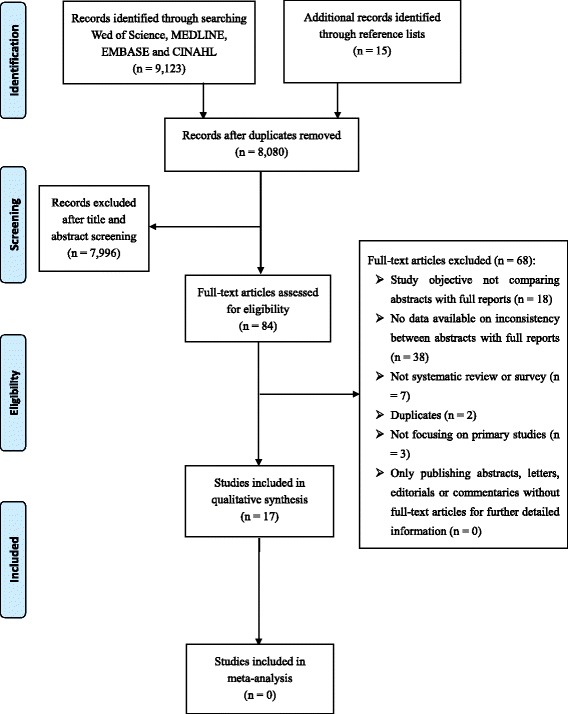
Study flow diagram showing the study selection process

There were 9123 records retrieved from the electronic databases. After removing duplicates and having screened the titles and abstracts, a total of 84 studies remained for full-text article assessment (kappa = 0.85, 95% confidence interval [CI]: 0.79 - 0.91 for record screening). Of these, 16 studies met the eligibility criteria [2–4, 7, 10–12, 18–26] and one additional study [27] was identified from the reference lists, yielding17 studies that were included for the data collection and analyses (kappa = 0.65, 95% CI: 0.57 - 0.75 for data extraction). Figure 1 presents the flow diagram of the study selection process.

Table 1 summarizes the characteristics of the included studies, with three systematic reviews [3, 12, 19] and fourteen surveys [2, 4, 7, 10, 11, 18, 20–27]. Eleven studies compared conference abstracts with their subsequent full reports [2, 7, 19–27], while the others investigated inconsistency between the abstract section and the main text in the same publication [3, 4, 10–12, 18]. Three studies reported that their primary studies were mostly from North America or Europe [2, 19, 24]. The median sample size in the primary studies ranged from 5 to 452. Three studies reported that they received academic funding to support their surveys [4, 11, 21]. Study quality was evaluated for the three systematic reviews, in which their scores on AMSTAR were 8 (out of 9) [19], 7 [12] and 6 [3], respectively. None of these three reviews provided information on conflict of interest for either the systematic reviews or each of the included primary studies [3, 12, 19]. Two studies did not consider the grey literature in their search strategies [3, 12]. Because there was no information available (such as protocol, ethics approval, registration) on the a priori research question and inclusion criteria before the conduct of the review, one study scored zero on the AMSTAR item 1 (“Was an '*a priori*' design provided?”) [3].

**Table 1:** General characteristics of included systematic reviews or surveys

First author, publication year	Study design	Field of study	Data sources for abstracts	Data sources for full reports	Study search frame	Numbers of included abstracts/full reports	Study country of primary studies	Study sample size in primary studies
*Abstracts followed by subsequent texts within the same publications (n = 6)*								
Boutron 2010 [4]	Survey	Composite fields	Medline via PubMed	Same as abstracts	Full reports: 2006 December	72/72	NR	For full reports: Median 84 (range: 4 to 6848)
Harris 2002 [18]	Survey	Psychology	Eight American Psychological Association journals^a^	Same as abstracts	From years 1997 to 1998	400/400	NR	NR
Lehmen 2014^b^ [12]	Systematic review	Spinal studies	Three spinal journals^c^	Same as abstracts	From years 2001 to 2010	40/40	NR	NR
Ochodo 2013^d^ [3]	Systematic review	Diagnostic accuracy studies	Journals with an impact factor of 4 or higher	Same as abstracts	Between January and June 2010	126/126	NR	For full reports: Median 151 (range: 12 to 20,765)
Pitkin 1999 [10]	Survey	General medicine	Five major general medical journals^e^ and a consecutive sample of articles published in the CMAJ	Same as abstracts	For the five journals: Jul 1, 1996-Jun 30, 1997; For CMAJ: Jul 1 1996-Aug 15, 1997	264/264	NR	NR
Ward 2004 [11]	Survey	Pharmacological studies	Six pharmacy-specific journals^f^	Same as abstracts	From June 2001 to May 2002	243/243	NR	NR
*Abstracts in conferences and meetings (n = 11)*								
Bhandari 2002 [2]	Survey	Orthopedics	The 1996 scientific program of the sixty-third Annual Meeting of the American Academy of Orthopaedic Surgeons	Medline and PubMed	Abstracts: 1996; Full reports: 1996-2001	465/159	For full reports: Most in North America (93.1%)	For full reports: Median 49 (range: 2 to 8141)
Davies 2002 [22]	Survey	Perinatology	The first annual Perinatal Society of Australia and New Zealand Congress in 1997	Medline	Abstracts: 1997; Full reports: up to Oct 2000	172/83	NR	NR
Dyson 2006 [27]	Survey	Veterinary anesthesiology	Conference abstracts from 1990 to 1999 annual meetings of the American College of Veterinary Anesthesiologists	Entrez PubMed and CAB Direct	Abstracts: 1990 - 1999; Full reports: 1990 - 2006	283/201	NR	NR
Hopewell 2006 [7]	Survey	Oncology	American Society of Clinical Oncology annual conference in 1992	The Cochrane Central Register of Controlled Trials and PubMed	Abstracts: 1992; Full reports: up to 2002	209/37	NR	For full reports: Median 120 (range: 8 to 612)
Klassen 2002 [23]	Survey	Pediatrics	The proceedings from the Society for Pediatric Research	PubMed, EMBASE, Cochrane Library, CINAHL, Web of Science, Current Contents, and HEALTHSTAR	Abstracts: 1992 - 1995; Full reports: up to July 2000	447/264	NR	For full reports: Median 45 (interquartile range: 20 to 116)
Kottachchi 2010^g^ [19]	Systematic review	Inflammatory bowel diseases	All abstracts of Phase III randomized controlled trials in inflammatory bowel disease accepted at Digestive Disease Week	MedLine, PubMed, EMBASE, and Google Scholar	Abstracts: 1998 -2003; Full reports: 1997-2009	82/64	For full reports: Europe (53%, 34/64); North America (28%, 18/64); and others	For full reports: Mean 94 (SD: 96)
Preston 2006 [24]	Survey	Orthopedics	The annual meeting of the Orthopaedic Trauma Association	PubMed	Abstracts: 1994 - 1997; Full reports: up to 2005	254/137	For full reports: Most in North America (93%)	For full reports: Mean 121 (standard deviation: 179)
Rosmarakis 2005 [20]	Survey	Infectious diseases and microbiology	From the first session of 7 of 15 major research categories presented in the 1999 and 2000 Interscience Conference on Antimicrobial Agents and Chemotherapy	Index Medicus	Abstracts: 1999 - 2000; Full reports: from 1999 to 2004. March	190/51	NR	NR
Snedeker 2010 [21]	Survey	Veterinary pre-harvest or abattoir-level interventions against foodborne pathogens	Ten conferences/meetings^h^ which involved presentations on pre-harvest or abattoir-level food safety	Four databases: Agricola, CAB Abstracts, Web of Knowledge, and Scholar’s Portal	From years 1995 to 2004	59/59	NR	For full reports: median 5 (range 1 to 35)
Toma 2006 [25]	Survey	Cardiology	Proceedings booklets and related Web sites for the American College of Cardiology scientific meetings (1999-2002).	PubMed, MEDLINE, EMBASE, and the Cochrane Cochrane Central Register of Controlled Trials databases	Abstracts: 1999 - 2002; Full reports: up to 2005	148/148	NR	For full reports: Median 452 (interquartile range: 173 to 1715)
Turpen 2010 [26]	Survey	Urology	Annual meetings of the American Urological Association	PubMed	Abstracts: 1999 - 2002; Full reports: up to 2007	126/79	NR	NR

*NR* not reported, *N/A* not available

^a^Including *Health Psychology*; *Journal of Abnormal Psychology*; *Journal of Consulting and Clinical Psychology*; *Journal of Counseling Psychology*; *Journal of Family Psychology*; *Neuropsychology*; *Professional Psychology: Research and Practice*; and *Psychological Assessment*

^b^The score for this study was 7 (out of 9) points on AMSTAR (a measurement tool to assess systematic reviews)

^c^Including *Spine*; *The Spine Journal*; and *Journal of Spinal Disorders and Techniques*

^d^The score for this study was 6 (out of 9) points on AMSTAR

^e^Including *Annals of Internal Medicine*; *BMJ*; *JAMA*; *Lancet*; and *New England Journal of Medicine*

^f^Including *American Journal of Health-System Pharmacy*; *The Annals of Pharmacotherapy*; *The Consultant Pharmacist*; *Hospital Pharmacy*; *Journal of the American Pharmacists Association*; *and Pharmacotherapy: The Journal of Human Pharmacology and Drug Therapy*

^g^The score for this study was 8 (out of 9) points on AMSTAR

^h^Including *International Symposium on Veterinary Economics and Epidemiology*; *Conference of Research Workers in Animal Disease*; *International Symposium on Shiga Toxin (Verocytotoxin) – Producing Escherichia coli Infections*; *Society for Veterinary Epidemiology and Preventive Medicine*; *International Symposium on the Epidemiology and Control of Foodborne Pathogens in Pork*; *International Symposium on Food-Borne Salmonella in Poultry*; *American Society of Microbiologists Conference on Salmonella*; *North East Conference on Avian Diseases*; *International Association of Food Protection (formerly IAMFES) Annual Meeting*; and *Institute of Food Technologists Annual Meeting*

**Fig. 2:**
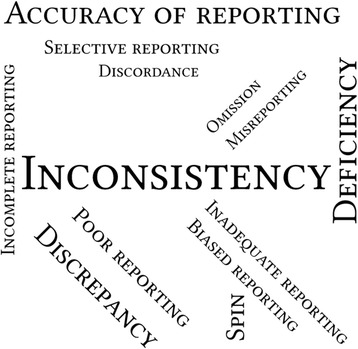
Word clouds of the terminologies used in the included studies, with the relative size of the terms in the word cloud corresponding to the frequency of their use

The most frequently used terminology to describe the abstract reporting problem were “inconsistency” (*n* = 14, out of 17 the included studies, 82%), “deficiency” (*n* = 11, 65%), “accuracy” (*n* = 10, 59%), and “discrepancy” (*n* = 8, 47%). Other terminology included “omission”, “misreporting”, “discordance”, “poor”, “biased”, “inadequate”, “incomplete”, and “selective reporting”, each of which appeared in at most4 studies. Figure 2 shows the word cloud of the terminologies used in the included studies.

Table 2 shows definitions, main findings and authors’ conclusions of inconsistency between abstracts and full reports in the included studies. The level of inconsistency ranged from 4% to 78%, with a median of 39% (interquartile range: 14% - 54%). In the studies that differentiated major from minor inconsistencies [2, 19, 20, 27], the level of major inconsistency ranged from 5% to 45% (median: 19%, interquartile range: 7% - 31%), which originated from the specification of the study design (5%) or sample size (37%), designation of a primary outcome measure (from 14% to 28%), presentation of main results (19%), or drawing a conclusion (6%). All the included studies concluded that abstracts were frequently inconsistently reported, and that efforts were needed to improve abstract reporting in primary biomedical research (Table 2).

**Table 2:** Definitions, main findings and authors’ conclusions of inconsistency between abstracts and full reports in the included studies

First author, publication year	Definition of inconsistency between abstracts and full reports	Main findings of inconsistent reporting	Authors’ conclusions
Bhandari 2002 [2]	Inconsistencies including *minor differences* (in study title, number of authors, presentation of all outcomes, and authors’ data interpretations) and *major differences* (in study objective and/or hypothesis, study design, primary or any secondary outcome measure, sample size, analyses, results, and precision measures.	Major inconsistency found in designating a primary outcome measure (14%), and results for primary outcome measure (19%)	The overall abstract-reporting quality was inadequate. The use of abstracts as a routine guide to orthopedic practice requires to be reconsidered.
Boutron 2010 [4]	In trials with primary outcome showing statistically non-significant results, the *spin* of reporting were (1) with a focus on statistically significant result; (2) with interpretation of non-significant results as showing equivalent or comparable treatment effectiveness; and (3) with an emphasis or claim of beneficial effect.	Spin identified in the abstracts of Results (38%) and Conclusions (58%) sections. Among the Conclusions section of abstracts, 24% focusing only on treatment effectiveness	Result reporting and interpretation in abstracts was frequently inconsistent with full reports in RCTs with non-significant findings.
Davies 2002 [22]	Abstracts were considered *discordant* with full reports if abstracts reported different sample size, or different primary aims and/or conclusions.	Discordance found in primary aims (25%), conclusions (35%) and sample sizes (39%).	Considerable differences found between abstracts and full reports in perinatology
Dyson 2006 [27]	*Major* differences defined as inconsistency on major results and conclusions; *minor* differences defined as inconsistency that would not change the overall clinical approach	Major differences existed in 7%, among which half of these inconsistencies could affect clinical action by changing the emphases of the conclusions.	Caution must be exercised in using information from conference abstracts in veterinary science
Harris 2002 [18]	Abstract rated as *deficient*: 1) if it contained information or a claim that was inconsistent with the body of the article (labeled discrepancy), 2) or if information or claim was reported in the abstract but not in the article (labeled omission)	Proportion of deficient abstracts ranged from 8% to 18% across journals, with an average of 13% over the entire sample	Readers should be aware that abstract-full-report inconsistencies are not uncommon in psychology.
Hopewell 2006 [7]	*Inconsistencies* defined as any differences in objectives, study designs, study quality, participants, interventions, outcomes, results, and conclusions.	16% of abstracts differed in primary outcomes, 54% in number of participants randomized and 78% in number of participants analyzed.	Information given in oncology conference abstracts is unstable and needed to be improved.
Klassen 2002 [23]	*Differences* in abstract-full-report pairs included conclusions, outcomes, effect sizes, and sample sizes.	5% of abstracts changed the conclusions regarding treatment efficacy, 13% had different effect sizes for outcomes, 59% had different sample sizes.	Significant differences between conference abstracts and subsequent full reports were found in pediatrics research.
Kottachchi 2010 [19]	Inconsistencies including *minor* (changes in number of authors and study title) and *major* (changes in study hypothesis/design, measurements in primary/ secondary outcomes, changes in sample size, statistical analysis, or different study results or measures of precision) inconsistency.	Minor change in number of authors (55%) and study title (70%). Major change in study design (5%), sample size (37%), primary outcome (28%), secondary outcome (31%), and conclusion (6%).	A substantial inconsistency was found when comparing abstracts with full reports in digestive diseases.
Lehmen 2014 [12]	Abstracts considered to have a *deficiency* if they had data that were inconsistent with or not found in full reports, or if they did not report pertinent negative results	75% of the abstracts had at least one 1 deficiency	A surprisingly high percentage of inconsistency between abstracts and full reports was reported in spinal RCTs.
Ochodo 2013 [3]	Abstracts defined as *overly optimistic* if they chose to report the best results only, or if they reported stronger recommendations or conclusions than in the full reports	23% of the abstracts were overly optimistic	Abstracts were frequently found to be misreported and overly optimistic in diagnostic accuracy studies.
Pitkin 1999 [10]	Abstracts considered *deficient* if they reported different data from full reports, or they provided data that could not be found in full reports	Deficient abstracts varied from 18% to 68%	Even in large-circulation general medical journals, data in abstracts were commonly inconsistency with full reports.
Preston 2006 [24]	*Inconsistencies* were discrepancies in study objective and/or hypothesis, study design, primary outcome measure, sample size, statistical analysis, results of primary/secondary outcomes, and conclusions.	29% abstract-full-report pairs had at least one inconsistency. Inconsistencies found in conclusions (7%), primary outcome measures (4%), sample size (18%), results for primary (8%) and secondary (29%) outcomes.	Inconsistencies were frequently observed. Most conclusions remained unchanged.
Rosmarakis 2005 [20]	Difference between abstracts and full reports categorized into *minor* or *major*; difference in any number by 10%, or statistically non-significant results changed to be significant (or vice versa) was considered major	Difference found in 59% pairs of abstracts and full reports, among which 77% was major difference	Significant inconsistencies were found between abstracts and full reports in infectious diseases and microbiology.
Snedeker 2010 [21]	*Difference* in abstract-paper(s) match including number of authors, study objectives, pathogen(s), intervention(s), species, sample size, housing, number of bacterial outcome measures, intervention effect, and overall conclusion	One-third (32%) of matches had different results; 14% differed in the direction of intervention effect; 26% significantly differed in outcome results; 11% differed in overall conclusion on efficacy of the intervention	Abstracts may not always accurately report the same information as in full reports in the field of pre-harvest and harvest-level food safety.
Toma 2006 [25]	*Inconsistencies* included differences in the study designs, purpose of trials, sources of funding, allocation concealment, sample size, results, and conclusions.	24% of abstracts had different sample size, 41% had different treatment effect estimates.	Inconsistencies between meeting abstracts and subsequent full reports were not uncommon in cardiology.
Turpen 2010 [26]	*Inconsistencies* included any differences in study design and results	29% abstract-full-report pairs had different numbers of participants randomized, 70% had unidentifiable primary outcome.	Abstract provided inconsistent results that could not allow urologists to critically appraise study validity.
Ward 2004 [11]	Abstracts considered *deficient* if they had data but not in full reports (omission), inaccurate factual information that differed from full reports, inconsistency in following the “Instructions for Authors” for respective journals, or difference in the information placement between abstracts and full reports	61% of the abstracts had at least one deficiency. 25% had an omission; 19% had qualitative inaccuracies; 25% had quantitative inaccuracies; 5% were inconsistent with the “Instructions for Authors”; 14% had information placement difference	Improvement is needed to rectify the inconsistency of abstract reporting in pharmacy-specific journals.

Table 3 shows the details on inconsistency for the study-validity-related factors between abstracts and full reports. Except for the research question or objective, intervention or exposure, study duration or design, and statistical analysis, inconsistencies were frequently reported in other factors of this type, with percentages of >10% in most cases. For instance, in the nine studies that assessed a total of 896 abstract-full-report pairs, conclusions in abstracts were found to be inconsistent with the full reports (ranging from 15% to 35%), or made stronger statements than in the full reports (17%). As presented in Table 4, three studies investigated factors related with inconsistent reporting between conference abstracts and full reports [2, 20, 21]. A longer time interval before publication of the full reports was found to be the only factor that was marginally or significantly related to an increased likelihood of reporting inconsistencies.

**Table 3:** Details on inconsistency for the study-validity-related factors between abstracts and full reports

Study-validity-related factor	Number of included studies (reference numbers)	Number of abstract-full-report pairs	Main findings of inconsistent reporting
Research question or objective	3 ([2, 19, 20])	274	Two studies reported high level (98% - 99%) of consistency for study objectives; One study found 10% difference in both study objectives and conclusions
Population or sample size	11 ([7, 12, 19–27]	1121	Sample sizes in abstracts were found to be smaller (9%), be different from full reports (17% - 78%), or have insufficient information on numbers of enrolled and analyzed participants/subjects (44% - 59%).
Intervention or exposure	1 ([21])	59	Full reports provided different/additional pathogens and/or interventions in two abstract-full-report pairs (3%).
Comparator	0	0	–
Outcome measure	8 ([2, 4, 7, 19, 22–24, 26])	647	It was found that inconsistency existed in designating a different primary outcome (4% - 28%), outcome measures were different (59%) between abstracts and full reports, or primary outcome was not stated in abstract (70% - 77%).
Study duration	1 ([20])	51	Sixteen abstracts (31%) reported different study period and/or population from full reports.
Study design	2 ([2, 19])	223	High level of consistency was found for study design (95% - 99%).
Statistical analysis	1 ([2])	159	Few abstracts (8%) reported the same statistical methods as in the full reports.
Result presentation	10 ([2, 3, 4, 12, 19–21, 24–26])	1131	Results in abstract were different from full reports (13% - 41%), with a statistically significant change leading to a change of study conclusion (6% - 32%), not reporting pertinent negative (40%) and pertinent positive (90%) findings, or selectively reporting favorable results (6%).
Result interpretation	5 ([3, 4, 7, 12, 21])	456	Result interpretation in abstracts was found to be inconsistent (4% - 15%), or overly optimistic (23%).
Conclusion or recommendation	9 ([3, 4, 12, 19, 21–24, 27])	896	Conclusions in abstracts were reported to be inconsistent (15% - 35%), or with stronger statements than in full reports (17%).

**Table 4:** Factors reported to be associated with inconsistent reporting between abstracts and full reports

First author, publication year	Study design	Field of study	Numbers of abstract-full-report pairs included for analyses	Factors related with inconsistent reporting	Association between factors and inconsistency
Bhandari 2002 [2]	Survey	Orthopedics	159	Time from abstract presentation to the publication of the full report	Longer time to publication of full reports significantly increased the likelihood of an inconsistency (odds ratio = 1.5 for per-month increase, *p* < 0.01)
Rosmarakis 2005 [20]	Survey	Infectious diseases and microbiology	51	Time from abstract presentation to publication of full reports	A trend found between longer time to publication of full reports and increased inconsistency (odds ratio = 1.76 for per year of delay, *p* = 0.07)
Snedeker 2010 [21]	Survey	Veterinary pre-harvest or abattoir-level interventions against foodborne pathogens	59	Time from abstract presentation to publication of full reports	Longer time to publication related with fewer outcome measures in full reports (than in abstracts) (*p* = 0.03)

## Discussion

In this scoping review assessing inconsistency between abstracts and full reports, we summarized the evidence from systematic reviews and surveys to show the literature mapping for the inconsistent abstract reporting in primary biomedical research. Abstract reports were frequently different from their corresponding full reports, with a high level of inconsistency. The length of time between the appearance of conference abstracts and the publication of full reports was the only factor reported to be associated with inconsistent reporting.

Readers usually rely on an initial assessment of an abstract in deciding whether to access the full report, draw conclusions about the study, or even make their decisions, especially when a full report is not available [4, 28]. For instance, Bhandari et al. found that over 50% of the chapters in the latest editions of some most influential orthopedic textbooks referenced at least one conference abstract, and these abstracts would be frequently cited in lectures and rounds [2]. Therefore, given their potential impact, all the summary information in abstracts should, at a minimum, be accurate and consistent with their full reports. However, abstracts are frequently prepared with the least care [1, 3].Our current review found a high level of inconsistency between abstracts and full reports, especially with respect to sample sizes, outcome measures, result presentation and interpretation, and conclusions or recommendations (Table 3). Unlike the included individual studies that evaluated a specific research area, or a group of journals or diseases, our review summarized all the available evidence from systematic reviews and surveys in various areas of the biomedical literature, and we consistently found severe problems in abstract reporting in the primary biomedical community. More efforts are warranted to reverse and prevent the inconsistency of abstract reporting.

There were two studies that also assessed the spin in the abstract reporting, in which the spin existed in the studies with overall non-significant results but with an overly-optimistic abstract that tried to claim significant results or strong recommendations [3, 4]. The spin may not always be relevant to our objective of identifying inconsistency between abstracts and full reports, because the spin could be the same in content and magnitude in both the abstract and the full report. However, when there is an attempt to incorrectly convince the audiences of a favorable finding or conclusion, the existence of spin in abstracts may pose a threat to distorting study findings and misleading readers of the biomedical literature, especially when readers do not go on to refer to the full reports for the study results in detail.

Many journals have adopted a policy of requiring structured abstracts, because they have been shown to be more informative, have greater readability, and be of better quality [29, 30]. However, one study has argued that the problem of inconsistent abstract reporting will not be mitigated by using structured abstracts [10]. In contrast, if structured abstracts inappropriately emphasize their main points, the inaccurate information that they convey could have a stronger impact on the biomedical community. For instance, some structured abstracts used spin to over-emphasize favorable effects in subgroups of patients, for secondary outcomes or in deliberately modified populations, or they made over-optimistically strong conclusions and recommendations, which would further mislead the audience [3, 4]. Furthermore, word count limitations in structured abstracts can sometimes cause key information to be omitted [21]. The effect of guideline checklists on improved consistent abstract reporting remains unknown, with sparse evidence available in the literature. The CONSORT (Consolidated Standards of Reporting Trials) guideline for abstracts, that was published to aid in improving structured abstract reporting for RCTs [31], might not prevent subtle inconsistencies, especially if editorial staff do not refer to full reports for painstaking scrutiny [10]. Similarly, one trial provided instructions on ensuring data accuracy in abstracts to authors, but found that this was ineffective in actually improving abstract reporting [32].

Some included studies explored the interpretations of the inconsistencies in conference abstract reporting. For example, given that some conference abstracts were presented when studies were ongoing or at an early stage, sample sizes in full reports would probably be updated from the preliminary results described in abstracts [20, 21]. However, one study argued that the inconsistencies may be deliberate, because authors avoided providing details or explanations of the inconsistencies (such as differently handling patients who were lost to follow-up or withdrawal, or adding more exclusion criteria) to achieve more favorable results in full reports [2]. Furthermore, in order to show significant findings in the full reports, authors may selectively report favorable findings, or deliberately change the way of defining primary outcomes, presenting and interpreting results or drawing conclusions [2, 19, 21, 23]. Three studies reported that having a longer time before the publication of full reports was associated with increased risk of inconsistent abstract reporting (Table 4) [2, 20, 21]. This might be partly explained if delayed publications had experienced several rejections from journals, and if authors then consciously or subconsciously modified their full reports to cater to the subsequent peer-review processes [20]. Some delays in publishing were due to having an extended study duration. In long duration studies, large amounts of data may be collected, which may then yield different statistical analysis results from the preliminary results as presented in the conference abstracts [2, 33].

To reduce or prevent inconsistency between abstracts and full reports, we recommend that the authors, reviewers and editorial staff should carefully scrutinize the consistency and accuracy of abstract reporting during the submission and peer-review processes [34]. Copyediting and proofreading should be performed strictly to avoid any confusion or inconsistency in abstracts after submissions are accepted. Journals may also consider more flexible word counts in structured abstracts to allow more details to be presented. Moreover, guidance and/or checklists are needed to facilitate authors, reviewers and editorial staff with their prompt assessment of inconsistency between abstracts and full reports. For conference abstracts, one might argue that conferences or meetings should require a publication-ready manuscript for their abstract submission [24]. However, this expectation is probably unrealistic and its impact remains largely unknown. In contrast, we recommend that editorial staff and reviewers should refer to the previously presented conference abstracts during the peer-review process, and authors should provide explanations of any inconsistencies between those abstracts and the full reports.

Our scoping review has some limitations. We limited the search to articles in English, which would omit studies in other languages. As most included studies focused on RCTs and/or conference abstracts, the findings from non-randomized studies, basic science and/or comparisons between abstract sections and main texts in the same publications, remained largely unknown. Also, we could not assess the quality of included surveys because no such validated guidance was available. Similarly, lack of information on the factors associated with inconsistent reporting between the abstract section and main text in the same publications restricted our investigations and recommendations in this area.

## Conclusion

In this scoping review of the state of abstract reporting in primary biomedical research, we found that abstracts were frequently inconsistent with full reports, based on evidence from systematic reviews and surveys in the literature. Efforts are needed to improve the consistency of abstract reporting in the primary biomedical community.

## Abbreviations

AMSTAR: a measurement tool to assess systematic reviews
CI: confidence interval
CONSORT: Consolidated Standards of Reporting Trials
EQUATOR: Enhancing Quality and Transparency in Health Research
PRISMA: Preferred Reporting Items for Systematic Reviews and Meta-Analyses
RCT: randomized controlled trial
